# *Trans*-Cinnamaldehyde, Eugenol and Carvacrol Reduce *Campylobacter jejuni* Biofilms and Modulate Expression of Select Genes and Proteins

**DOI:** 10.3389/fmicb.2019.01837

**Published:** 2019-08-07

**Authors:** Basanta R. Wagle, Abhinav Upadhyay, Indu Upadhyaya, Sandip Shrestha, Komala Arsi, Rohana Liyanage, Kumar Venkitanarayanan, Dan J. Donoghue, Annie M. Donoghue

**Affiliations:** ^1^Department of Poultry Science, University of Arkansas, Fayetteville, AR, United States; ^2^Department of Animal Science, University of Connecticut, Storrs, CT, United States; ^3^School of Agriculture, Tennessee Tech University, Cookeville, TN, United States; ^4^Department of Chemistry and Biochemistry, University of Arkansas, Fayetteville, AR, United States; ^5^Poultry Production and Product Safety Research Unit, United States Department of Agriculture – Agriculture Research Station, Fayetteville, AR, United States

**Keywords:** *C. jejuni*, biofilm, phytochemicals, inhibition, inactivation, gene/protein expression

## Abstract

*Campylobacter jejuni* is the leading cause of human foodborne illness globally, and is strongly linked with the consumption of contaminated poultry products. Several studies have shown that *C. jejuni* can form sanitizer tolerant biofilm leading to product contamination, however, limited research has been conducted to develop effective control strategies against *C. jejuni* biofilms. This study investigated the efficacy of three generally recognized as safe status phytochemicals namely, *trans*-cinnamaldehyde (TC), eugenol (EG), or carvacrol (CR) in inhibiting *C. jejuni* biofilm formation and inactivating mature biofilm on common food contact surfaces at 20 and 37°C. In addition, the effect of phytochemicals on biofilm architecture and expression of genes and proteins essential for biofilm formation was evaluated. For the inhibition study, *C. jejuni* was allowed to form biofilms either in the presence or absence of sub-inhibitory concentrations of TC (0.75 mM), EG (0.61 mM), or CR (0.13 mM) for 48 h and the biofilm formation was quantified at 24-h interval. For the inactivation study, *C. jejuni* biofilms developed at 20 or 37°C for 48 h were exposed to the phytochemicals for 1, 5, or 10 min and surviving *C. jejuni* in the biofilm were enumerated. All phytochemicals reduced *C. jejuni* biofilm formation as well as inactivated mature biofilm on polystyrene and steel surface at both temperatures (*P* < 0.05). The highest dose of TC (75.64 mM), EG (60.9 mM) and CR (66.56 mM) inactivated (>7 log reduction) biofilm developed on steel (20°C) within 5 min. The genes encoding for motility systems (*flaA*, *flaB*, and *flgA*) were downregulated by all phytochemicals (*P* < 0.05). The expression of stress response (*cosR*, *ahpC*) and cell surface modifying genes (*waaF*) was reduced by EG. LC-MS/MS based proteomic analysis revealed that TC, EG, and CR significantly downregulated the expression of NapA protein required for oxidative stress response. The expression of chaperone protein DnaK and bacterioferritin required for biofilm formation was reduced by TC and CR. Scanning electron microscopy revealed disruption of biofilm architecture and loss of extracellular polymeric substances after treatment. Results suggest that TC, EG, and CR could be used as a natural disinfectant for controlling *C. jejuni* biofilms in processing areas.

## Introduction

*Campylobacter* is one of the major foodborne pathogens that causes gastroenteritis in humans worldwide (Marder et al., 2017). Recent reports have shown that the incidence of *Campylobacter* infections has increased by 10% in 2017 compared to 2014–2016 with an annual incidence of 17.83 per 100,000 people in the United States (Marder et al., 2017). Among the major *Campylobacter* species, *Campylobacter jejuni* is responsible for approximately 90% of the reported campylobacteriosis cases in humans (Cody et al., 2013). In addition, *C. jejuni* infections have been associated with the occurrence of Guillain-Barré syndrome and reactive arthritis causing significant economic losses and disease burden globally (Spiller, 2007; Gradel et al., 2009; Hoffmann et al., 2012).

The primary source of human *C. jejuni* infections is the handling and/or consumption of contaminated poultry products (Rosner et al., 2017). The survival of *C. jejuni* in the environment such as water and feed plays a critical role in *C. jejuni* colonization in the birds and subsequent contamination of poultry products during carcass processing (Annan-Prah and Janc, 1988; Dhillon et al., 2006; Boysen et al., 2016). Several studies have shown that the biofilm-forming capacity in bacteria facilitates increased tolerance to sanitizers (Somers et al., 1994; Trachoo and Frank, 2002; Trachoo et al., 2002; Melo et al., 2017). Therefore, there is a high possibility that product contamination in processing environment could be due to the presence of biofilms. A biofilm is an assemblage of surface-associated microbial communities embedded within the matrix of extracellular polymeric substances (Donlan, 2002; Donlan and Costerton, 2002). The ability of *C. jejuni* to form biofilm has been demonstrated on different surfaces, including plastic, glass and steel under different oxygen concentrations (Trachoo et al., 2002; Reuter et al., 2010; Bronowski et al., 2014; Brown et al., 2014; Bronnec et al., 2016). Moreover, studies have demonstrated that *C. jejuni* biofilm formation can be enhanced by atmospheric oxygen and in the presence of chicken meat juice (Reuter et al., 2010; Brown et al., 2014). Thus, *C. jejuni* biofilms constitute a significant food safety hazard.

The formation of bacterial biofilm begins with initial attachment of bacteria to a surface. The surface attachment strengthens, and the bacterial community becomes irreversibly attached to the target surface. This is followed by maturation of the biofilm and dispersion of bacteria to new location (Donlan and Costerton, 2002). A number of genes that contribute to biofilm formation have been characterized in *C. jejuni* (Bronowski et al., 2014). Several genes coding for motility (Joshua et al., 2006; Kalmokoff et al., 2006; Kim et al., 2015) are essential for biofilm formation. These include *flaA, flaB, flaC, flaG, fliA, fliS, flgA*, and *flhA*. In addition, genes encoding stress response (*spoT, csrA, ahpC, cosR*, and *cprS*) (Fields and Thompson, 2008; McLennan et al., 2008; Svensson et al., 2009; Oh and Jeon, 2014; Turonova et al., 2015), bacterial cell surface modifications (*peb4, waaF*) (Asakura et al., 2007; Naito et al., 2010) and quorum sensing (*luxS*) (Reeser et al., 2007) are also critical for biofilm formation and maturation in *C. jejuni*. One of the critical components of bacterial biofilm is the exopolysaccharide layer (EPS), which protects underlying bacterial population from harsh environmental conditions. The impermeability of EPS along with the slower growth rates and metabolism of bacteria in the biofilms makes them resistant to disinfectants, antimicrobials and antibiotics (Reuter et al., 2010; Borges et al., 2016). In a recent study, *C. jejuni* population in biofilms was found to exhibit up to 32-fold higher resistance to gentamicin than in the corresponding planktonic forms (Malik et al., 2017). In addition, biofilm facilitates *C. jejuni* to survive for a longer period of time (up to 24 days) as compared to planktonic cells under aerobic conditions or in water (Joshua et al., 2006; Lehtola et al., 2006). Thus, developing appropriate processing plant hygiene and sanitation are critical for controlling *C. jejuni* biofilms.

Current intervention approaches for controlling *C. jejuni* biofilms include use of chemicals (Somers et al., 1994; Trachoo et al., 2002; Melo et al., 2017), biofilm-degrading enzymes (Brown et al., 2015; Kim et al., 2017) and application of bacteriophages (Siringan et al., 2011). Chemical disinfectants such as chlorine, trisodium phosphate and quaternary ammonium compounds have been extensively investigated for their antibiofilm efficacy against *C. jejuni*, however, these compounds have limited effectiveness in controlling *C. jejuni* biofilm, especially in the presence of organic matter (Northcutt et al., 2005; Oyarzabal, 2005). Moreover, production of mutagens is of concern (Dore, 2015). The use of biofilm-degrading enzymes for controlling biofilms has been reported in various bacteria, including *C. jejuni* (Brown et al., 2015; Kim et al., 2017). However, the efficacy of biofilm-degrading enzyme could be reduced with the production of high quantities of EPS and proteolytic activity of exoenzymes produced by the mature biofilms (Whitchurch et al., 2002). Similarly, treatment of *C. jejuni* biofilms with bacteriophages has limited application due to the chance of emergence of resistance strains (Siringan et al., 2011). Therefore, there is a need for a novel strategy to control *C. jejuni* biofilms.

Phytochemicals have been used as natural antimicrobials for treating human infections since ancient time. The majority of phytochemicals are secondary metabolites produced as a defense mechanism to protect plants from pathogenic microorganisms (Borges et al., 2016). A variety of phytochemicals have been evaluated for their antibacterial effect against foodborne pathogens and several active components have been identified (Burt, 2004; Holley and Patel, 2005). However, very few studies have investigated the potential of phytochemicals for controlling *C. jejuni* biofilms. *Trans*-cinnamaldehyde (TC) is an aldehyde extracted from the bark of cinnamon (*Cinnamomum zeylandicum*), whereas eugenol (EG) and carvacrol (CR) are the active components of clove oil (*Eugenia caryophyllus*) and oregano oil (*Origanum glandulosum*), respectively. All the aforementioned phytochemicals are classified as generally recognized as safe by the United States Food and Drug Administration (21 Code of Federal Regulation part 172.515) (Adams et al., 2004, 2005; Knowles et al., 2005). Previous studies have demonstrated the antibiofilm effect of TC, EG and CR in various bacteria such as *Salmonella* Typhimurium (Trevisan et al., 2018), *S*. Enteritidis (Čabarkapa et al., 2019), *Listeria monocytogenes* (Upadhyay et al., 2013) and *Pseudomonas aeruginosa* (Letsididi et al., 2018; Lou et al., 2019). However, their antibiofilm effect in *C. jejuni* has not been determined.

In this study, we investigated the antibiofilm efficacy of TC, EG, and CR against *C. jejuni* at two temperatures (20 and 37°C) and on two surfaces (polystyrene and stainless-steel) that are commonly encountered in processing plant environment. The effect of phytochemical treatments on *C. jejuni* biofilm architecture was visualized using scanning electron microscopy and confocal laser scanning microscopy. Moreover, the effects of TC, EG, and CR on the transcription of *C. jejuni* genes and proteins critical for biofilm formation were determined.

## Materials and Methods

### *C. jejuni* Strain and Culture Conditions

*Campylobacter jejuni* NCTC 11168 strain was cultured in 10 mL of *Campylobacter* enrichment broth (CEB; International Diagnostics Group, Bury, Lancashire, United Kingdom) and incubated under microaerophilic condition (5% O_2_, 10% CO_2_, and 85% N_2_) at 42°C for 48 h. Following growth, *C. jejuni* was centrifuged and washed twice with Butterfield’s phosphate diluent (BPD, 0.625 mM potassium dihydrogen phosphate, pH 7.2) and resuspended in CEB to use as inoculum.

### Preparation of Chicken Meat Juice

A previously published method was used for the preparation of chicken meat juice (Birk et al., 2004). Briefly, frozen whole chickens were obtained from the University of Arkansas poultry pilot processing plant (Fayetteville, AR, United States) and thawed overnight at 4°C. The meat juice was collected and centrifuged at 4,000 rpm for 20 min to remove debris followed by filter sterilization (0.2 μm cellulose acetate membrane; VWR International, United States). Based on published literature (Brown et al., 2014) and growth curve analysis, chicken meat juice was added to CEB at 5% level and used for biofilm experiments.

### Determination of *C. jejuni* Biofilm Formation on Polystyrene Plates and Stainless-Steel Coupons

The biofilm formation of *C. jejuni* on polystyrene plates and steel coupons was determined according to a previously published method with slight modifications (Brown et al., 2014). Briefly, 200 μL of CEB broth containing *C. jejuni* (∼6.0 Log CFU) was added to 96-well polystyrene plates or on steel coupons kept in 24-well polystyrene plates and incubated for 48 h at 20 or 37°C under aerobic condition to facilitate biofilm formation. The biofilm formation was determined by 2, 3, 5-Triphenyltetrazolium chloride (TTC) staining at 24 h intervals. After staining, TTC solution was removed followed by air-drying and bound TTC dye was dissolved in 20% acetone in ethanol, and the A_500_ value of the solution was measured. A similar procedure was used to determine *C. jejuni* biofilm formation in broth containing 5% chicken meat juice.

### Determination of Sub-Inhibitory Concentrations and Minimum Bactericidal Concentrations of Phytochemicals

The sub-inhibitory concentration (SIC) and minimum bactericidal concentration (MBC) of each phytochemical against *C. jejuni* was determined according to a previously described method (Upadhyay et al., 2017b; Wagle et al., 2017a,b). All three phytochemicals were purchased from Sigma-Aldrich (St. Louis, MO, United States). In brief, twofold dilutions of TC, EG, or CR in CEB (100 μL/well) were made in sterile 96-well polystyrene plates (Costar, Corning Incorporated, Corning, NY, United States) followed by inoculation with equal volume of *C. jejuni* (10^6^ CFU/mL) and incubated at 37°C for 24 h under microaerophilic conditions. The growth of *C. jejuni* was determined by plating on *Campylobacter* Line Agar (CLA) plates (Line, 2001). The highest concentration of phytochemicals that did not inhibit bacterial growth was selected as the SIC for the study, whereas the lowest concentration of phytochemicals that reduced *C. jejuni* counts significantly (>2 Log_10_ CFU/mL) was taken as MBC.

### Biofilm Inhibition and Inactivation Assays on Polystyrene Plates

The ability of TC, EG, and CR in inhibiting *C. jejuni* biofilm formation on polystyrene plates was determined according to a previously published method (Reeser et al., 2007; Lu et al., 2012). Two hundred microliters of culture (∼6.0 Log CFU) was added to each well of a sterile 96-well polystyrene plate, followed by addition of SICs of TC, EG, or CR. The plates were incubated at 20 or 37°C for 48 h. A similar procedure was followed to test the antibiofilm efficacy of phytochemicals in the presence of 5% chicken meat juice. The biofilm formation was determined at 24 h intervals. At each time point, the spent medium was removed and the well was gently washed three times with BPD. The bacteria in the biofilms were removed using cell scrapper and plated on CLA plates. The biofilm associated *C. jejuni* were enumerated after incubation at 42°C for 48 h.

The inactivation of mature *C. jejuni* biofilms by TC, EG and CR was determined as described previously (Kim et al., 2017). Briefly, *C. jejuni* (∼6.0 Log CFU, 200 μL) was allowed to form biofilm in 96-well polystyrene plate at 20 or 37°C for 48 h. After mature biofilm was formed, the inactivation was carried out with 200 μL of TC (18.91, 37.82, or 75.64 mM), EG (15.22, 30.45, or 60.90 mM) or CR (16.64, 33.28, or 66.56 mM) in BPD for 1, 5, or 10 min. The treatment solution was discarded and 200 μL of Dey-Engley neutralizing broth (Difco Laboratories, Sparks, MD, United States) was added. The number of surviving *C. jejuni* in the biofilm was determined as described above. The study was repeated in the presence of 5% chicken meat juice.

### Preparation of Stainless-Steel Coupons

A previously described method (Jeong and Frank, 1994) was used for the preparation of stainless-steel coupons (Type 304; diameter 5/8 inch; no. 4 finish). Briefly, steel coupons were cleaned with acetone followed by washing in distilled water and soaking in 100% ethanol. Finally, steel coupons were rinsed with distilled water, subjected to air dry and autoclaved at 121°C for 15 min.

### Biofilm Inhibition and Inactivation Assays on Stainless-Steel Coupons

To determine the effect of TC, EG, and CR in inhibiting biofilm formation and inactivating mature biofilm on stainless-steel, a published method was used with slight modifications (Trachoo et al., 2002). For the inhibition study, steel coupons were incubated with 1 mL of *C. jejuni* (∼6.0 Log CFU) in 24-well polystyrene plates containing SICs of phytochemicals at 20 or 37°C for 48 h. *C. jejuni* counts in the biofilms on steel coupons were determined after washing three times with BPD at 24 h intervals.

For the inactivation of mature biofilm on steel coupons, mature biofilm was developed on steel coupons placed in 24-well polystyrene plates containing *C. jejuni* (∼6.0 Log CFU) at 20 or 37°C for 48 h. After biofilm formation, steel coupons were rinsed three times with BPD and transferred to new polystyrene plates and exposed to various doses of TC (18.91, 37.82, or 75.64 mM), EG (15.22, 30.45, or 60.90 mM), or CR (16.64, 33.28, or 66.56 mM) for 1, 5, or 10 min. Following rinsing with BPD, the steel coupons were placed in 50 mL centrifuged tubes containing 3 × *g* sterile glass beads (diameter 2 mm; Thermo Fisher Scientific, Carlsbad, CA, United States) and 10 mL of Dey-Engley neutralizing broth, and vortexed for 1 min. The solution was serially diluted and plated on CLA. *C. jejuni* counts were enumerated after incubation of plates at 42°C for 48 h. Similar inhibition and inactivation studies were conducted in the presence of 5% chicken meat juice in broth medium.

### Microscopic Examination of *C. jejuni* Biofilms

Environmental scanning electron microscopy (ESEM) and confocal laser scanning microscopy (CLSM) were used to visualize the effect of TC, EG, and CR on biofilm architecture and the viability of *C. jejuni* in biofilms. *C. jejuni* (6 Log CFU) was inoculated on stainless-steel coupons and Lab-Tek two-chamber (no. 1) borosilicate coverglass system (Nunc, Rochester, NY, United States) for ESEM and CLSM, respectively to develop biofilms at 37°C for 2 days, and were exposed to TC (18.91 mM), EG (15.22 mM), or CR (16.64 mM) for 10 min. All the samples were rinsed with BPD before further processing. For ESEM, samples were fixed with 2.5% glutaraldehyde in 0.1 M PIPES buffer for 1 h, as described previously (Brown et al., 2014). After fixation, samples were rinsed three times with PIPES buffer and dehydrated in a series of ethanol solutions (at 30, 40, 50, 60, 70, 80, 90, and three times at 100%) for at least 10 min for each step. The biofilms were dried and coated with gold using Emitech SC7620 sputter coater (Quorum Technologies, Ltd., East Sussex, United Kingdom) for 135 s. The coated biofilm samples were visualized using SE detectors at 10 kV beam (Philips XL30 ESEM, FEI Company, Hillsboro, OR, United States). For the CLSM, the viability of *C. jejuni* in the biofilms were determined using FilmTracer^TM^ Live/Dead Biofilm Viability Kit (Molecular probes, Eugene, OR, United States) according to a published method (Asakura et al., 2007). SYTO-9 and propidium iodide stains were used for the differential staining of live and dead cells. After staining for 20 min, biofilms were visualized in each chamber using a hybrid detector at 63 × objective in the Leica SP5 Confocal microscope (Leica Microsystems Inc., Buffalo Grove, IL, United States).

### Gene Expression Analysis of *C. jejuni* Exposed to Phytochemical Treatments

The effect of TC, EG, and CR on the transcription of *C. jejuni* genes essential for biofilm formation was determined using real-time quantitative PCR (RT-qPCR) (Upadhyay et al., 2017b; Wagle et al., 2017a,b). Briefly, *C. jejuni* (∼6.0 Log CFU/mL) was incubated in the presence or absence of SICs of TC, EG or CR at 37°C for 12 h. The total RNA was extracted using RNA mini kit (Invitrogen, Carlsbad, CA, United States) and complementary DNAs were prepared using iScript cDNA synthesis kit (Bio-Rad Laboratories, Inc., CA, United States). The primers were designed using Primer 3 Software (National Center for Biotechnology Information, Bethesda, MD, United States) and obtained from Integrated DNA Technologies, Inc. (Coralville, IA, United States) (Table 1). The specificity of primer was tested using NCBI-Primer BLAST, melt curve analysis and *in silico* PCR (Bikandi et al., 2004). The amplified products were detected by using SYBR Green reagents (Bio-Rad Laboratories, Inc.). The 16s rRNA gene was used as the endogenous control and comparative critical threshold (ΔΔCt) method was employed to analyze relative expressions of candidate genes on Quant Studio 3 real-time PCR system (Applied Biosystems, Thermo Fisher Scientific).

**TABLE 1:** Primers used for gene expression analysis using real-time quantitative PCR.

**Gene with Accession no.**	**Primer**	**Sequence (5′-3′)**
16S-rRNA (NC_002163.1)	Forward Reverse	5′-ATAAGCACCGGCTAACTCCG-3′ 5′-TTACGCCCAGTGATTCCGAG-3′
*flaA* (NC_002163.1) (NC_002163.1)	Forward Reverse	5′-AGCGTTTGCAAAACCTGTGG-3′ 5′-ATGAGTAGCGCAGGAAGTGG-3′
*flaB* (NC_002163.1) (NC_002163.1)	Forward Reverse	5′-AGCGTTTGCAAAACCTGTGG-3′ 5′-ATGAGTAGCGCAGGAAGTGG-3′
*flaG* (NC_002163.1) (NC_002163.1)	Forward Reverse	5′-AGAACAAGTGAGACACAGGCT-3′ 5′-TTGCTGTCCATCATCGCCTT-3′
*flgA* (NC_002163.1) (NC_002163.1)	Forward Reverse	5′-TTTGCACGAATCCTTTCGCC-3′ 5′-TCGGGGTTTTAAGCGAAGCA-3′
*peb4* (NC_002163.1) (NC_002163.1)	Forward Reverse	5′-AAGGTGGTGAGCTTGGTTGG-3′ 5′-TTAAGCGCGAAAGCAGCATC-3′
*waaF* (NC_002163.1) (NC_002163.1)	Forward Reverse	5′-CCTGGTGCAAGCTTTGGAAG-3′ 5′-TTGTTCGGCTTTTCCTGCAC-3′
*cosR* (NC_002163.1) (NC_002163.1)	Forward Reverse	5′-TCAGGTTCTTCCCAGATGGC-3′ 5′-CGCACTTAGCAAGACATTCGG-3′
*ahpC* (NC_002163.1) (NC_002163.1)	Forward Reverse	5′-AGTTCGCCATGCTGTGGTTA-3′ 5′-CCTGCAGGACAAACTTCACC-3′
*luxS* (NC_002163.1) (NC_002163.1)	Forward Reverse	5′-AGTGTTGCAAAAGCTTGGGA-3′ 5′-GCATTGCACAAGTTCCGCAT-3′

### Proteomic Analysis of *C. jejuni* in Biofilms Exposed to Phytochemical Treatments

The effect of TC, EG, and CR on the proteome of *C. jejuni* in the biofilms was determined using liquid chromatography with tandem mass spectrometry (LC-MS/MS) as described previously (Miyamoto et al., 2015). Briefly, *C. jejuni* (∼6.0 Log CFU/mL) was incubated in the presence or absence of SICs of phytochemicals and allowed to develop biofilms at 37°C for 48 h. Following washing with BPD buffer, proteins were extracted using B-Per^®^ bacterial protein extraction reagent (Thermo Fisher Scientific) and subjected to SDS-PAGE (4–12% Bis-Tris protein gel, Thermo Fisher Scientific). Each lane of gel was excised and destained with 50% acetonitrile in ammonium bicarbonate (Thermo Fisher Scientific) for 45 min and vacuum dried for 10 min. The protein extracts were treated with dithiothreitol (1.5 mg/mL in 25 mM ammonium bicarbonate; Bio-Rad) and reduced with iodoacetamide (37 mg/mL in ammonium bicarbonate; Bio-Rad) for 1 h. Following removal of iodoacetamide, the proteins were digested with trypsin (20 ng per μL in 25 mM ammonium bicarbonate) and incubated overnight at 37°C. The resultant peptides were analyzed by LC-MS/MS technique using Agilent 1200 series microflow HPLC coupled to a Bruker AmaZon-SL quadrupole ion trap mass spectrometer (Bruker Daltonics Inc., Billerica, MA, United States) with a captive spray ionization source. The proteins were identified by matching MS/MS spectra to protein sequences of *C. jejuni* available at the uniprot.org using in house MASCOT software (Matrix Science Inc., Boston, MA, United States) (Perkins et al., 1999). The proteins were identified based on <5% false discovery rate using at least 2 unique peptides from a protein.

### Statistical Analyses

A completely randomized design was used in the study with duplicate samples and the study was repeated three times. The data for each treatment and control were pooled from three independent trials within the same study before analysis. Bacterial counts were logarithmic transferred to maintain the homogeneity of variance (Byrd et al., 2001). The data of inhibition and inactivation assays were analyzed by using PROC MIXED procedure in the SAS version 9.3 software (SAS Institute Inc., Cary, NC, United States) and the treatment means were separated by least-square means analysis at *P* < 0.05 for statistical difference. The gene expression data were analyzed by using PROC MIXED procedure in the SAS and Student’s *t*-test was used for comparisons between treatment and controls. For the proteomic analysis, Scaffold Proteome Software version 4.8 (Proteome Software Inc., Portland, OR, United States) was used to analyze MASCOT files and differentially expressed proteins between treated and un-treated biofilms were determined using Student’s *t*-test.

## Results

### Sub-Inhibitory Concentrations and Minimum Bactericidal Concentrations of TC, EG, and CR Against *C. jejuni*

The SICs of phytochemicals were determined based on growth curve analysis (data not shown). We observed that 0.75 mM (0.01% v/v) of TC, 0.61 mM (0.01% v/v) of EG and 0.13 mM (0.002% v/v) of CR were the highest concentration of phytochemicals that did not reduce the growth of *C. jejuni* and were selected as the respective SIC for the study. Similarly, the lowest concentrations that reduced *C. jejuni* counts significantly were 2.27 mM (0.03%) for TC, 1.83 mM (0.03%) for EG and 0.26 mM (0.004%) for CR and these doses were taken as MBC.

### Effect of Chicken Juice on *C. jejuni* Biofilm Formation on Polystyrene Plates and Stainless-Steel Coupons

**FIGURE 1:**
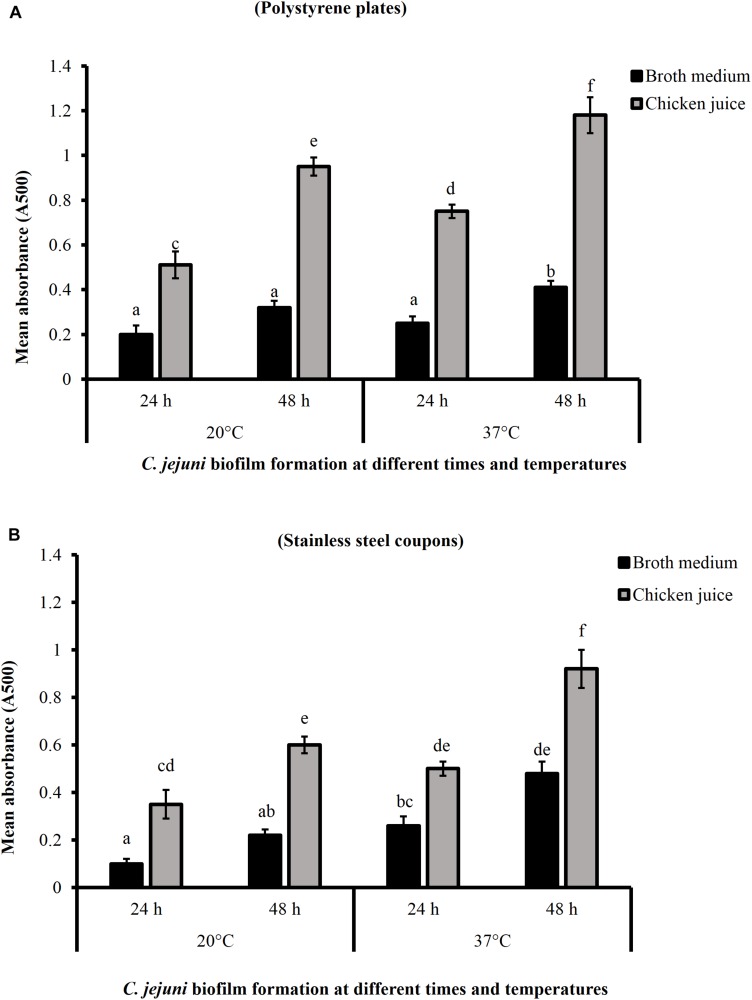
*Campylobacter jejuni* biofilm formation on polystyrene plates **(A)** and stainless-steel coupons **(B)** at 20 and 37°C. Error bars represent SEM (*n* = 6). *C. jejuni* (∼6.0 Log CFU/mL) in the presence or absence of 5% chicken meat juice was incubated to form biofilm. The biofilm formation was determined by TTC staining at 24 h interval. Different letters indicate the statistical difference across time or temperatures (*P* < 0.05).

Figure 1 shows the *C. jejuni* biofilm formation on polystyrene plates and stainless-steel coupons in the presence or absence of 5% chicken meat juice. The presence of chicken juice significantly enhanced *C. jejuni* biofilm formation by ∼ twofold at both temperatures on both surfaces. In addition, the absorbance (indicator of adhered and metabolically active *C. jejuni*) was significantly higher in the 48 h biofilms developed at 37°C than at 20°C.

### Effect of Sub-Inhibitory Concentrations of TC, EG, and CR on *C. jejuni* Biofilm Formation on Polystyrene Plates and Stainless-Steel Coupons

**FIGURE 2:**
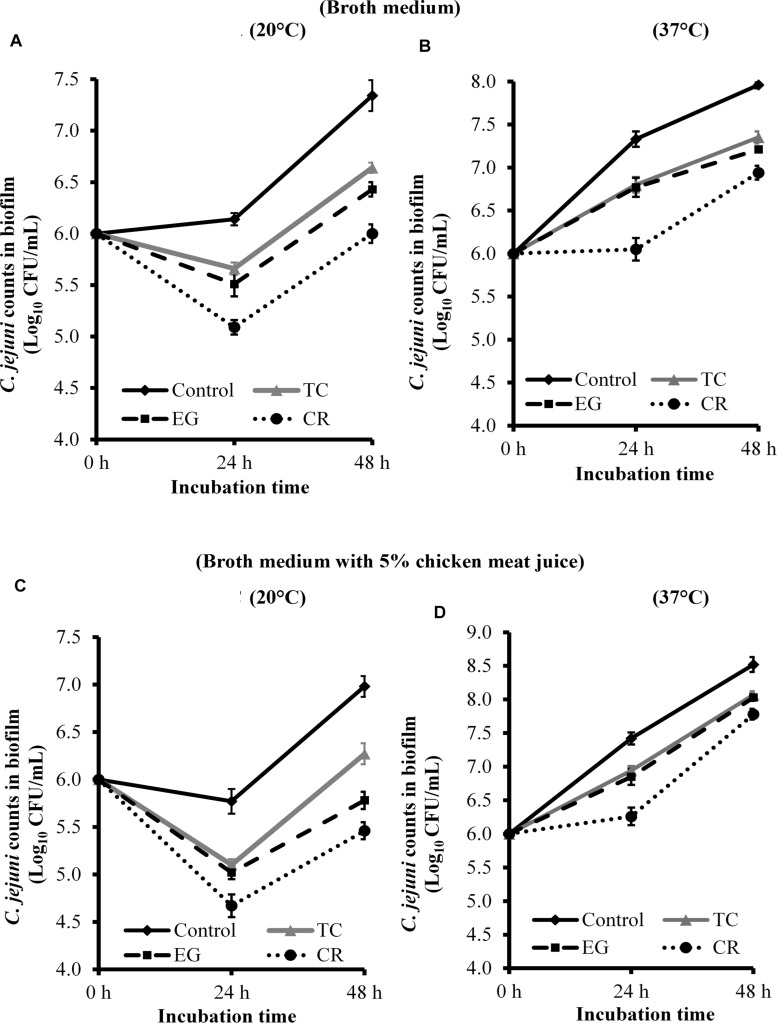
Effect of SICs of *trans*-cinnamaldehyde (TC), eugenol (EG), and carvacrol (CR) on *Campylobacter jejuni* NCTC 11168 biofilm formation on polystyrene plates in the broth medium **(A,B)** and in the presence of 5% chicken meat juice **(C,D)**. Error bars represent SEM (*n* = 6). *C. jejuni* (∼6.0 Log CFU/mL) in the presence of TC (0.75 mM), EG (0.61 mM), or CR (0.13 mM) was incubated to form biofilm in sterile 96-well plates at 20°C **(A,C)** or 37°C **(B,D)**. The number of *C. jejuni* in the biofilm was enumerated at 24 or 48 h. All treatments were significantly different from control at both 24 and 48 h (*P* < 0.05).

Figure 2 presents the effect of TC, EG, and CR on *C. jejuni* biofilm formation on polystyrene plates at 20 and 37°C in the presence and absence of chicken juice. *C. jejuni* biofilm developed in broth medium had ∼7.3 and 8 Log CFU/mL of pathogen count at 20°C (Figure 2A) and 37°C (Figure 2B), respectively, after 48 h of incubation. The presence of SICs of TC, EG, and CR significantly reduced *C. jejuni* counts in the biofilm developed at 20°C by ∼0.5 and 0.7 Log CFU/mL, respectively, at 24 and 48 h as compared to the control. At 37°C, the reduction was ∼0.56 Log CFU/mL at both time points (*P* < 0.05). Similar results were observed in the presence of chicken meat juice at both temperatures (Figures 2C,D) where the three phytochemicals exerted an inhibitory effect and reduced *C. jejuni* biofilm formation by ∼0.5 Log CFU/mL. Carvacrol was the most effective treatment and reduced *C. jejuni* in the biofilm by ∼1.5 and ∼0.75 Log CFU/mL, respectively, at 20°C and 37°C at the end of 48 h as compared to respective controls (Figures 2C,D).

**FIGURE 3:**
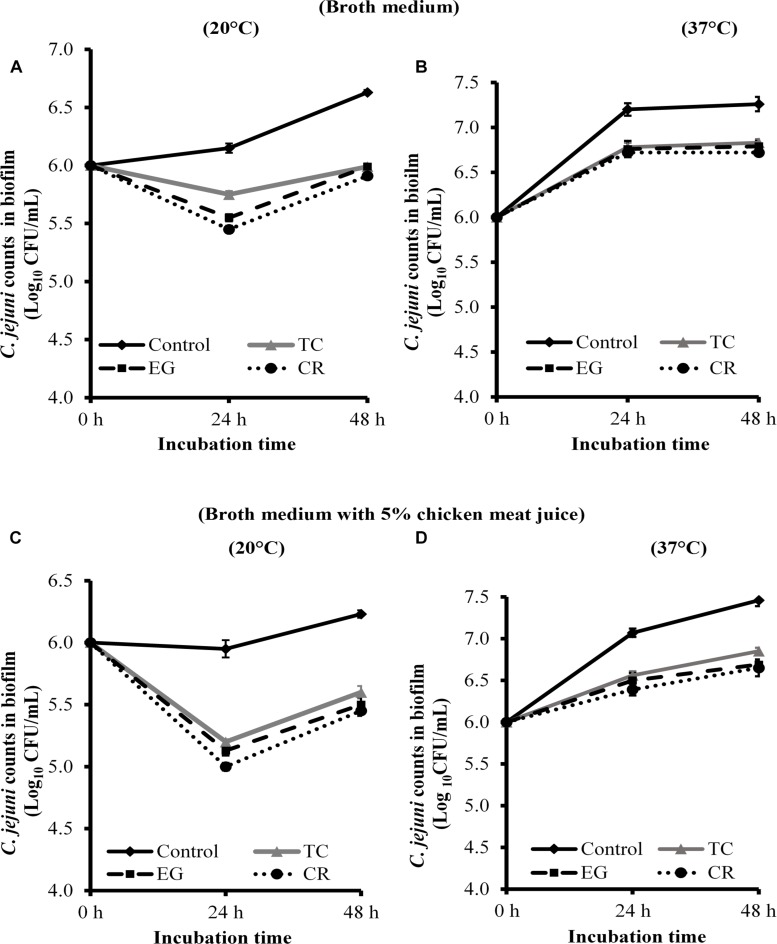
Effect of SICs of *trans*-cinnamaldehyde (TC), eugenol (EG), and carvacrol (CR) on *Campylobacter jejuni* NCTC 11168 biofilm formation on stainless-steel coupons in the broth medium **(A,B)** and in the presence of 5% chicken meat juice **(C,D)**. Error bars represent SEM (*n* = 6). *C. jejuni* (∼6.0 Log CFU/mL) in the presence of TC (0.75 mM), EG (0.61 mM) or CR (0.13 mM) was incubated to form biofilm on steel coupons at 20°C **(A,C)** or 37°C **(B,D)**. The number of *C. jejuni* in the biofilm was enumerated at 24 or 48 h. All treatments were significantly different from control at both 24 and 48 h (*P* < 0.05).

The effect of TC, EG, and CR in inhibiting *C. jejuni* biofilm formation on stainless-steel coupons is shown in Figure 3. All phytochemicals reduced *C. jejuni* in the biofilm by ∼0.6 and ∼0.45 Log CFU/mL, respectively, at 20 (Figure 3A) and 37°C (Figure 3B) at both time points (*P* < 0.05). Similar reductions were observed when biofilm was developed in the presence of 5% chicken meat juice on steel coupons (Figures 3C,D). Although phytochemicals were effective in reducing *C. jejuni* counts as compared to respective controls at 24 and 48 h, the phytochemicals did not inhibit the growth of *C. jejuni* biofilm at 48 h as compared to 24 h except at 37°C in CEB (Figure 3B).

### Effect of TC, EG, and CR on Mature *C. jejuni* Biofilms Developed on Polystyrene Plates and Stainless-Steel Coupons

The efficacy of TC, EG and CR in inactivating mature *C. jejuni* biofilms on polystyrene plates and steel coupons is shown in Tables 2, 3, respectively. The number of *C. jejuni* recovered from control (biofilms not subjected to treatments) on polystyrene plates was ∼7.8 and ∼8.4 Log CFU/mL, respectively, at 20 and 37°C in the broth medium (Table 2). Similar counts were observed in the presence of 5% chicken meat juice on polystyrene plates (*P* > 0.05). At 20°C on polystyrene, highest doses of TC (75.64 mM; 1%), EG (60.90 mM; 1%), and CR (66.56 mM; 1%) reduced the counts of *C. jejuni* to below detection limit (reductions > 7.0 Log CFU/mL) in the broth medium within 1 min of exposure time. Lower doses of TC (18.91 and 37.82 mM, corresponds to 0.25 and 0.5%, respectively), EG (15.22 and 30.45 mM; 0.25 and 0.5%), and CR (16.64 and 33.28 mM; 0.25 and 0.5%) reduced *C. jejuni* in the biofilms in a range from 3.2 to 3.8 Log CFU/mL in 10 min. The phytochemicals were effective in inactivating *C. jejuni* biofilms in the presence of chicken juice as well. For example, in the presence of chicken juice at 20°C, 66.56 mM CR reduced *C. jejuni* counts by 6.69 Log CFU/mL (most effective treatment) followed by 33.28 mM CR or 60.90 mM EG (reductions ∼3.8 Log CFU/mL) in 10 min exposure time (Table 2). At 37°C in the broth medium, 60.90 mM EG or 66.56 mM CR was the most effective in reducing *C. jejuni* counts by ∼3.84 Log CFU/mL at the end of 10 min. The reductions were similar in the presence of chicken juice where TC (75.64 mM), EG (60.90 mM), and CR (66.56 mM) reduced the counts by 1.64, 4.11, and 4.88 Log CFU/mL, respectively, in 10 min (Table 2).

**TABLE 2:** Effect of *trans*-cinnamaldehyde (TC; 18.91, 37.82, 75.64 mM), eugenol (EG; 15.22, 30.45, 60.90 mM), and carvacrol (CR; 16.64, 33.28, 66.56 mM) on mature *Campylobacter jejuni* NCTC 11168 biofilm formed on polystyrene microtiter plates at 20°C or 37°C in the broth medium and in the presence of chicken meat juice (*n* = 6).

		**20°C**						**37°C**					
		**Broth medium**			**Broth + 5% chicken juice**			**Broth medium**			**Broth + 5% chicken juice**		
**Treatments**		**1 min**	**5 min**	**10 min**	**1 min**	**5 min**	**10 min**	**1 min**	**5 min**	**10 min**	**1 min**	**5 min**	**10 min**
**Control**		8.10 ± 0.10^a*^	7.79 ± 0.08^a^	7.78 ± 0.08^a^	7.81 ± 0.05^a^	7.81 ± 0.05^a^	7.81 ± 0.05^a^	8.35 ± 0.11^a^	8.44 ± 0.10^a^	8.39 ± 0.09^a^	8.59 ± 0.04^a^	8.66 ± 0.03^a^	8.63 ± 0.04^a^

**TC (mM)**	18.91	6.80 ± 0.09^b^	5.63 ± 0.08^b^	4.54 ± 0.07^b^	6.69 ± 0.11^b^	6.15 ± 0.04^b^	5.14 ± 0.04^b^	7.80 ± 0.05^b^	7.78 ± 0.05^b^	7.54 ± 0.04^b^	8.38 ± 0.06^ab^	8.26 ± 0.03^b^	7.98 ± 0.04^b^
	37.82	6.41 ± 0.07^c^	4.32 ± 0.09^c^	4.00 ± 0.06^cd^	6.45 ± 0.14^b^	5.19 ± 0.06^c^	4.89 ± 0.03^bc^	7.62 ± 0.05^bc^	7.53 ± 0.04^b^	7.39 ± 0.07^b^	7.67 ± 0.04^d^	7.68 ± 0.06^c^	7.60 ± 0.14^c^
	75.64	ND^e^	ND^d^	ND^e^	5.27 ± 0.06^cd^	4.89 ± 0.06^c^	4.59 ± 0.07^c^	7.68 ± 0.12^b^	5.79 ± 0.09^d^	5.61 ± 0.07^d^	7.52 ± 0.03^d^	7.36 ± 0.05^c^	6.99 ± 0.19^d^

**EG (mM)**	15.22	6.58 ± 0.05^bc^	5.40 ± 0.09^b^	4.52 ± 0.06^b^	6.68 ± 0.09^b^	6.20 ± 0.05^b^	5.00 ± 0.07^bc^	7.49 ± 0.06^bc^	6.47 ± 0.49^c^	6.34 ± 0.10^c^	8.09 ± 0.08^bc^	7.66 ± 0.16^c^	6.75 ± 0.16^de^
	30.45	6.09 ± 0.07^d^	4.34 ± 0.05^c^	3.91 ± 0.09^d^	6.38 ± 0.04^b^	5.11 ± 0.05^c^	4.82 ± 0.02^c^	7.57 ± 0.11^bc^	5.59 ± 0.10^d^	4.95 ± 0.16^e^	7.84 ± 0.25^cd^	6.16 ± 0.04^e^	5.72 ± 0.07^f^
	60.90	ND^e^	ND^d^	ND^e^	5.01 ± 0.06^de^	4.89 ± 0.03^c^	3.93 ± 0.08^d^	7.31 ± 0.08^c^	5.16 ± 0.21^e^	4.55 ± 0.27^fg^	6.67 ± 0.38^e^	5.18 ± 0.11^f^	4.52 ± 0.20^g^

**CR (mM)**	16.64	6.37 ± 0.07^cd^	5.25 ± 0.11^b^	4.33 ± 0.07^bc^	5.44 ± 0.01^c^	5.16 ± 0.04^c^	4.80 ± 0.08^c^	6.94 ± 0.17^d^	6.30 ± 0.11^c^	6.07 ± 0.05^c^	6.91 ± 0.16^e^	6.76 ± 0.05^d^	6.46 ± 0.09^e^
	33.28	6.14 ± 0.07^cd^	4.26 ± 0.09^c^	3.98 ± 0.07^cd^	4.73 ± 0.09^e^	4.49 ± 0.05^d^	4.03 ± 0.11^d^	5.87 ± 0.15^e^	5.55 ± 0.05^d^	4.89 ± 0.09^ef^	5.41 ± 0.12^f^	5.18 ± 0.05^f^	4.50 ± 0.09^g^
	66.56	ND^e^	ND^d^	ND^e^	3.30 ± 0.32^f^	3.11 ± 0.07^e^	1.12 ± 0.26^e^	5.76 ± 0.09^e^	5.07 ± 0.25^e^	4.46 ± 0.09^g^	4.90 ± 0.07^g^	4.77 ± 0.13^g^	3.75 ± 0.13^h^

The biofilms were exposed to phytochemical treatment for 1, 5, or 10 min. Values (Log CFU/mL) presented as mean ± standard error of the mean. ^*^The different superscripts within columns differ significantly at *P* < 0.05. ND: not detectable below 1 Log CFU/mL.

**TABLE 3:** Effect of *trans*-cinnamaldehyde (TC; 18.91, 37.82, 75.64 mM), eugenol (EG; 15.22, 30.45, 60.90 mM), and carvacrol (CR; 16.64, 33.28, 66.56 mM) on mature *Campylobacter jejuni* NCTC 11168 biofilms formed on stainless-steel coupons at 20°C or 37°C in the broth medium and in the presence of chicken meat juice (*n* = 6).

**20°C**								**37°C**					
		**Broth medium**			**Broth + 5% chicken juice**			**Broth medium**			**Broth + 5% chicken juice**		
**Treatments**		**1 min**	**5 min**	**10 min**	**1 min**	**5 min**	**10 min**	**1 min**	**5 min**	**10 min**	**1 min**	**5 min**	**10 min**
**Control**		6.38 ± 0.11^a*^	6.24 ± 0.09^a^	6.35 ± 0.05^a^	6.08 ± 0.07^a^	6.00 ± 0.04^a^	6.05 ± 0.03^a^	7.91 ± 0.10^a^	7.84 ± 0.03^a^	7.88 ± 0.05^a^	7.79 ± 0.08^a^	7.89 ± 0.09^a^	7.75 ± 0.07^a^

**TC (mM)**	18.91	3.62 ± 0.08^b^	3.34 ± 0.06^b^	1.85 ± 0.08^b^	4.80 ± 0.07^b^	4.23 ± 0.04^b^	3.65 ± 0.06^b^	6.11 ± 0.08^b^	5.70 ± 0.10^b^	5.05 ± 0.08^b^	6.43 ± 0.04^b^	6.12 ± 0.05^b^	5.80 ± 0.07^b^
	37.82	1.77 ± 0.04^c^	ND^c^	ND^c^	2.11 ± 0.12^c^	1.56 ± 0.22^c^	ND^c^	5.21 ± 0.05^c^	5.00 ± 0.07^c^	4.15 ± 0.07^cd^	5.96 ± 0.08^c^	5.71 ± 0.04^c^	5.31 ± 0.03^c^
	75.64	ND^d^	ND^c^	ND^c^	1.10 ± 0.20^d^	ND^e^	ND^c^	5.01 ± 0.06^c^	4.30 ± 0.08^d^	3.85 ± 0.07^d^	5.55 ± 0.07^d^	5.29 ± 0.08^d^	4.94 ± 0.12^d^

**EG (mM)**	15.22	1.59 ± 0.09^c^	ND^c^	ND^c^	2.00 ± 0.11^c^	1.02 ± 0.17^d^	ND^c^	5.76 ± 0.03^b^	4.95 ± 0.08^c^	4.33 ± 0.06^c^	6.19 ± 0.09^bc^	5.58 ± 0.07^cd^	5.13 ± 0.09^cd^
	30.45	ND^d^	ND^c^	ND^c^	1.03 ± 0.13^d^	ND^e^	ND^c^	4.13 ± 0.09^d^	3.06 ± 0.09^e^	1.10 ± 0.06^f^	5.33 ± 0.05^d^	3.26 ± 0.07^e^	1.88 ± 0.15^e^
	60.90	ND^d^	ND^c^	ND^c^	ND^e^	ND^e^	ND^c^	ND^f^	ND^f^	ND^g^	4.14 ± 0.15^f^	3.20 ± 0.05^e^	ND^f^

**CR (mM)**	16.64	ND^d^	ND^c^	ND^c^	1.80 ± 0.26^c^	1.21 ± 0.19^d^	ND^c^	3.21 ± 0.04^e^	2.94 ± 0.10^e^	1.95 ± 0.10^e^	4.71 ± 0.06^e^	3.39 ± 0.27^e^	2.25 ± 0.16^e^
	33.28	ND^d^	ND^c^	ND^c^	ND^e^	ND^e^	ND^c^	ND^f^	ND^f^	ND^g^	2.58 ± 0.06^g^	1.66 ± 0.36^f^	ND^f^
	66.56	ND^d^	ND^c^	ND^c^	ND^e^	ND^e^	ND^c^	ND^f^	ND^f^	ND^g^	ND^h^	ND^g^	ND^f^

The biofilms were exposed to phytochemical treatments for 1, 5, or 10 min. Values (Log CFU/mL) presented as mean ± standard error of the mean. ^*^The different superscripts within columns differ significantly at *P* < 0.05. ND: not detectable below 1 Log CFU/mL.

On stainless-steel coupons, *C. jejuni* counts in the control biofilms developed at 20°C were ∼6.3 Log CFU/mL (Table 3). The biofilms developed at 37°C had ∼7.8 Log CFU/mL of *C. jejuni* present. In the biofilms developed at 20°C in the broth medium, EG (30.45, 60.90 mM) or CR (16.64, 33.28, and 66.56 mM) reduced the counts of *C. jejuni* to below detection limit as early as 1 min of treatment time. TC (37.82, 75.64 mM) treatments reduced the counts to below detection limit within 5 min of treatment. The lowest dose (15.22 mM) of EG also reduced the counts below detection within 5 min (reductions > 6.25 Log CFU/mL) whereas 18.91 mM TC significantly reduced the counts by ∼4.5 Log CFU/mL in 10 min exposure time. In the presence of 5% chicken meat juice, the lowest dose of TC (18.91 mM) reduced *C. jejuni* counts significantly by 2.4 Log CFU/mL and higher doses of TC (37.82 or 75.64 mM) reduced counts below detection within 10 min. The counts were also reduced below detection by all three doses of EG and CR.

At 37°C in the broth medium, 33.28 or 66.56 mM CR or 60.90 mM EG was the most effective and reduced the counts below detection limit in 1 min. In addition, TC at 18.91, 37.82, and 75.64 mM reduced *C. jejuni* counts by 2.8, 3.7, and 4 Log CFU/mL, respectively, in 10 min exposure time. Similar results were observed in the presence of chicken juice where 33.28 or 66.56 mM of CR and 60.90 mM EG were the most effective treatments and reduced *C. jejuni* counts by ∼7.7 Log CFU/mL. In addition, the antibacterial activities of the TC, EG, and CR were significantly increased with an increase in exposure time on steel coupons (*P* < 0.05). For example, at 37°C in the presence of chicken juice, 16.64 mM CR had significantly a higher reduction in 10 min than in 1 min exposure time (reductions ∼5.5 vs. 3 Log CFU/mL).

### Effect of Phytochemicals on Mature Biofilms Architecture and Viability of *C. jejuni* in the Biofilms

**FIGURE 4:**
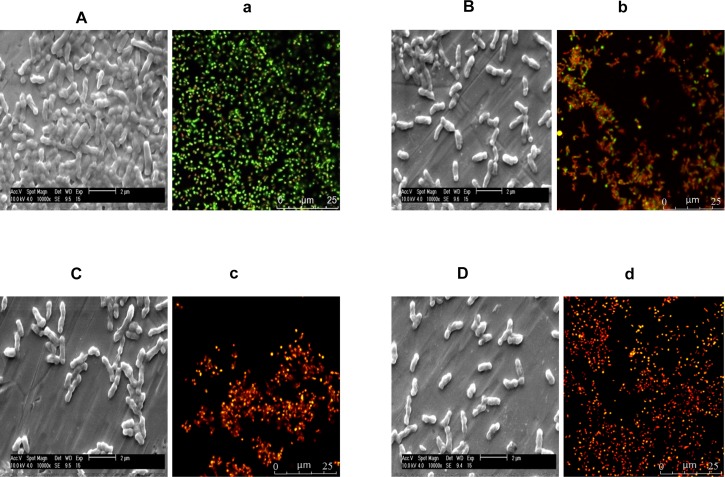
Environmental scanning electron microscopy (ESEM) and confocal laser scanning micrographs (CLSM) of *Campylobacter jejuni* NCTC 11168 biofilm before treatment with phytochemicals (controls; **A,a**) and after treatment with 18.91 mM of *trans*-cinnamaldehyde **(B,b)**, 15.22 mM eugenol **(C,c)**, or 16.64 mM carvacrol **(D,d)**. *C. jejuni* (6 Log CFU) was inoculated on stainless-steel coupons and Lab-Tek two-chamber (no. 1) borosilicate coverglass system for ESEM and CLSM, respectively, to develop biofilms at 37°C for 2 days. The biofilms were exposed to phytochemicals for 10 min followed by gentle washing. For the ESEM **(A–D)**, the treated biofilms were fixed with 2.5% glutaraldehyde and dehydrated in a series of ethanol concentration (30–100%). Dried biofilms were coated with gold using Emitech SC7620 sputter coater and visualized using 10 kV beam in ESEM. For the CLSM **(a–d)**, the treated biofilms were stained with 0.01 mM SYTO (green dye) and 0.06 mM propidium iodide (red) for 20 min, and visualized at 63 × objective in Leica SP5 confocal microscope.

The effect of TC, EG, and CR on the biofilm architecture and viability of *C. jejuni* in the biofilms was visualized using ESEM and CLSM (Figure 4). The biofilm structure was intact and covered with EPS in control *C. jejuni* biofilm not exposed to phytochemicals (Figure 4A), whereas the exposure to TC (18.91 mM), EG (15.22 mM), and CR (16.64 mM) for 10 min removed a majority of the biofilm structure as depicted by loss of EPS and scattering of *C. jejuni* cells (Figures 4B–D, respectively). In addition, confocal microscopy revealed that the majority of *C. jejuni* were live (stained green) in the control biofilms, and dead (stained red) after treatments with TC, EG, and CR for 10 min (Figures 4a–d).

### Effect of Phytochemicals on the Expression of *C. jejuni* Genes Coding for Biofilm Formation

**FIGURE 5:**
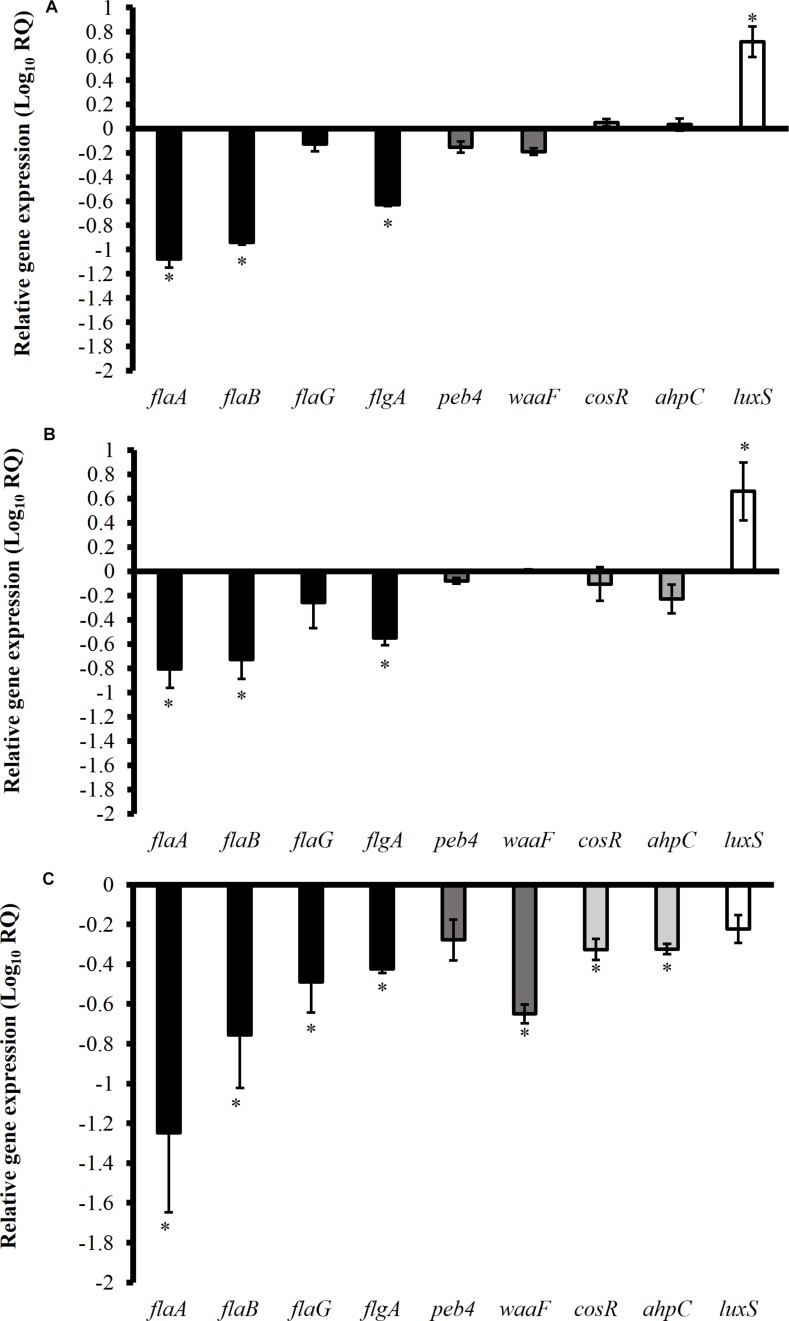
Effect of SICs of *trans*-cinnamaldehyde **(A)**, carvacrol **(B)**, and eugenol **(C)** on the expression of *Campylobacter jejuni* (NCTC 11168) genes critical for biofilm formation. Error bars represent SEM (*n* = 6). *C. jejuni* (∼6.0 Log CFU/mL) in the presence of TC (0.75 mM), EG (0.61 mM) or CR (0.13 mM) was incubated at 37°C for 12 h followed by RNA extraction and cDNA synthesis. RT-qPCR was conducted with 16S-rRNA serving as endogenous control. ^*^indicates significant change in the expression of genes at *P* < 0.05.

Figure 5 shows the effect of TC, EG, and CR on the expression of *C. jejuni* genes critical for biofilm formation. Phytochemicals at SICs level significantly modulated the expression of genes encoding for motility, cell surface modifications, stress response and quorum sensing. The SIC of TC significantly downregulated bacterial cell mobility genes *flaA*, *flaB*, and *flgA* by ∼11.7, 9, and 4.3-fold, respectively (Figure 5A). However, quorum sensing gene (*luxS*) responsible for cell to cell communication during biofilm formation was upregulated by ∼ sixfold (*P* < 0.05). The expression of stress response genes (*cosR*, *ahpC*) was not affected by TC treatment (*P* > 0.05). Similar to TC, CR also downregulated motility genes *flaA*, *flaB*, *flgA* and upregulated *luxS* (Figure 5B). The phytochemical EG downregulated (fold change > 2) majority of the tested genes (*flaA*, *flaB*, *flaG, flgA, waaF, cosR*, and *ahpC*) critical for *C. jejuni* biofilm formation (Figure 5C).

### Effect of Phytochemicals on *C. jejuni* Biofilm Proteome

Overall, 76 proteins were identified in the proteome of *C. jejuni* present in the biofilms. Table 4 shows the differential protein expression of *C. jejuni* in biofilms subjected to phytochemicals treatment as compared to control biofilms. The presence of SIC of TC significantly upregulated three proteins and downregulated three proteins critical for biofilm formation (*P* < 0.05). The upregulated proteins were flagellar protein (FliL), cytochrome c553 (Cyf) and putative peptidyl-prolyl *cis-*isomerase (Cbf2), whereas periplasmic nitrate reductase (NapA), chaperone (DnaK), and bacterioferritin were downregulated. Similar results were observed with EG, which upregulated cytochrome c553 (Cyf) and downregulated NapA (*P* < 0.05). The SIC of CR significantly upregulated NapB and FliL proteins and downregulated NapA and DnaK proteins. However, other identified proteins were not affected by the presence of phytochemicals (*P* > 0.05).

**TABLE 4:** List of identified proteins significantly altered in *C. jejuni* NCTC 11168 biofilms treated with TC, EG, or CR as compared to untreated (control) biofilms.

**Proteins name and function(s)**	**Alternative ID**	**Molecular weight (kDa)**	***P*-value**
**Upregulated with TC**			
Putative peptidyl-prolyl *cis-trans* isomerase	Cbf2	30	0.039
Cytochrome c553	Cyf	11	0.002
Flagellar protein FliL	FliL	20	0.047
**Downregulated with TC**			
Periplasmic nitrate reductase	NapA	105	0.002
Chaperone protein DnaK	DnaK	67	0.016
Bacterioferritin, putative	CJJ81176_1519	17	0.016
**Upregulated with EG**			
Cytochrome c553	Cyf	11	0.025
Uncharacterized protein	CJJ81176_0474	8	0.035
**Downregulated with EG**			
Periplasmic nitrate reductase	NapA	105	0.024
Uncharacterized protein	CJJ81176_0974	16	0.013
**Upregulated with CR**			
Periplasmic nitrate reductase, electron transfer subunit	NapB	19	0.047
Flagellar protein FliL	FliL	20	0.001
Uncharacterized protein	CJJ81176_1382	27	0.025
**Downregulated with CR**			
Periplasmic nitrate reductase	NapA	105	0.016
Chaperone protein DnaK	DnaK	67	0.016

## Discussion

*Campylobacter* contamination of poultry products represents a major risk factor for human campylobacteriosis. Despite being nutritionally fastidious, there is sufficient evidence that biofilm formation plays a critical role in the survival of *C. jejuni* in the processing environment (Murphy et al., 2006; García-Sánchez et al., 2017; Castro et al., 2018). The role of *C. jejuni* biofilm as a source of carcass contamination at the poultry processing facility is currently unknown especially since a comprehensive study determining *C. jejuni* biofilm at processing plant facility is lacking. Therefore, as a first step toward understanding the role of *C. jejuni* biofilms as a source of potential carcass contamination, we have developed *C. jejuni* biofilms on various surfaces commonly encountered in the processing plant and at conditions mimicking the processing plant environment. Furthermore, the efficacy of phytochemicals in killing *C. jejuni* biofilms was investigated.

The antibiofilm potential of phytochemicals was tested in the presence of 5% chicken meat juice to represent the meat environment as it has been shown previously that the presence of meat extracts modulates biofilm formation in *C. jejuni* (Brown et al., 2014). Brown et al. (2014) had also reported that chicken and pork meat juice (5–100%) enhanced *C. jejuni* biofilm formation by increasing attachment of *C. jejuni* to abiotic surfaces.

In order to effectively control *C. jejuni* biofilms in the processing plant, both prevention of biofilm formation and killing of pre-formed mature biofilms are important. Therefore, we tested the efficacy of phytochemicals in reducing biofilm formation as well as inactivating mature *C. jejuni* biofilms. We used the SIC of phytochemicals in the inhibition studies and hypothesized that the phytochemicals affect the critical genes and proteins required by planktonic cells for biofilm formation. The biofilms were developed for 48 h since our results (data not shown) and literature (Reeser et al., 2007) suggest that *C. jejuni* forms mature biofilm by 48 h. Carvacrol was most effective in inhibiting *C. jejuni* biofilms formation on polystyrene plates at 24 h (Figure 2), whereas these phytochemicals were not significantly different in their antibiofilm efficacy among on steel coupons (Figure 3) (*P* > 0.05). Similar results were reported with the SIC of TC, EG, and CR against *L. monocytogenes* (Upadhyay et al., 2013), where the authors observed significant reductions (∼1.5 Log CFU/mL) in counts in the biofilms developed for 48 h at 25 and 37°C. Although the phytochemicals were effective in reducing *C. jejuni* biofilm formation compared to respective control (Figures 2, 3), they did not inhibit the growth of *C. jejuni* biofilm at 48 h as compared to 24 h except at 37°C in CEB on steel coupons (Figure 3B). This could potentially be due to degradation of phytochemicals leading to reduced efficacy or microbial metabolism of these compounds. However, the exact cause of this observation is not known.

Previous studies have shown that phytochemicals at SICs level significantly modulate the expression of genes critical for virulence of various pathogenic bacteria (Qiu et al., 2010; Maisuria et al., 2016), including *C. jejuni* (Castillo et al., 2014; Kovács et al., 2016; Upadhyay et al., 2017b; Wagle et al., 2017a,b). However, the potential mechanism of action of TC, EG and CR against *C. jejuni* genes critical for biofilm formation has not been studied. Therefore, a gene expression study was performed to study the change in gene expression profile of *C. jejuni* in response to TC, EG and CR. A variety of genes critical for *C. jejuni* biofilm formation has been previously characterized (Bronowski et al., 2014). Bacterial genes encoding flagellins (FlaA, FlaB, and FlaG) and flagellar biosynthesis protein (FlgA) are necessary at the initial stage of *C. jejuni* biofilm formation (Kalmokoff et al., 2006; Kim et al., 2015). Previously, proteomic analysis revealed that flagellins proteins (FlaA, FlaB) were expressed at higher levels in *C. jejuni* biofilms than in planktonic cells (Kalmokoff et al., 2006). Moreover, *C. jejuni flgA* mutants were non-motile leading to reduced biofilm formation on food contact surfaces (Kim et al., 2015). Similarly, cell-binding protein (Peb4) and inner core of lipooligosaccharides (WaaF) protect the bacterial cell during stress and contribute to survival by forming biofilm (Asakura et al., 2007; Naito et al., 2010). It was previously reported that CosR is an essential response regulator in *C. jejuni*, which regulates the transcription of oxidative stress genes (*katA*, *ahpC*) (Hwang et al., 2012; Turonova et al., 2015). In addition, CosR is the key protein in the maturation of biofilm and its overexpression was reported to enhance biofilm formation in *C. jejuni* (Oh and Jeon, 2014). Likewise, quorum sensing or cell-to-cell signaling has been reported to play an important role in the cell attachment to form biofilm. Biofilm formation was significantly reduced in *C. jejuni luxS* mutants compared to wild-type (Reeser et al., 2007). Therefore, we selected all the aforementioned genes critical for *C. jejuni* biofilm formation. We observed that TC, EG, and CR at SICs significantly downregulated the expression of select flagellar genes critical for initial attachment during biofilm formation (Figure 5). However, these phytochemicals differ from one another in reducing expression of quorum sensing and stress response genes. For example, EG significantly downregulated *cosR* and *ahpC*, however, these genes were not affected by TC and CR. Moreover, quorum sensing gene *luxS* was upregulated by TC and CR indicating that it could be a bacterial compensatory mechanism against phytochemical treatments. These findings suggest that TC, EG, and CR may act through different mechanism(s).

To determine the effect of TC, EG, and CR on proteome of *C. jejuni* present in the biofilms, LS-MS/MS based protein identification and quantification of phytochemical-treated and un-treated *C. jejuni* biofilms was conducted. Periplasmic nitrate reductase (NapABC enzyme) is an enzyme responsible for utilization of nitrate as an energy source for bacterial growth and also protects against oxidative stress (Pittman et al., 2007). Similarly, heat shock protein 70 kD (also known as chaperone DnaK) contributes to motility, stress responses, and pathogenesis in *Escherichia coli* (Arita-Morioka et al., 2015). A loss of this protein lead to reduction in biofilm formation in *Staphylococcus aureus*. Similar findings were observed with *Streptococcus mutans* where it regulates RpoS and CsgD proteins essential for curli-dependent biofilm formation (Rockabrand et al., 1998; Arita-Morioka et al., 2015). In our proteomic analysis, NapA was significantly downregulated in TC, EG, and CR-treated biofilms as compared to un-treated *C. jejuni* biofilms (Table 4). The SICs of TC and CR also reduced the expression of DnaK. In addition, we identified a few uncharacterized proteins and the specific roles of such proteins need to be explored in future studies. These results varied from the effect of EG on proteome of *C. jejuni* planktonic cells where proteins contributing to motility (MotA, MotB, FliA, FliD, FliF, FliL, and FliY) and energy taxis (IlvH, CetA, and CetB) were downregulated (Upadhyaya et al., 2017). In a separate study, TC significantly down-regulated the expression of several proteins (FrdA, AhpC, PstS, CeuE, HemC, and AspA) that contribute to cellular metabolism and stress tolerance on *C. jejuni* planktonic cells (Upadhyay et al., 2017a). This variation could be due to the differential protein expression between planktonic and biofilm states of *C. jejuni* as reported by Kalmokoff et al. (2006). In addition, the differential protein/gene expression between two states of *C. jejuni* could potentially contribute to the discrepancy in results between gene expression and proteomic analysis as the gene expression study was conducted on planktonic cells in the present study. These findings suggest that antibiofilm effect of TC, EG, and CR could potentially be mediated through modulation of these proteins critical for *C. jejuni* biofilm formation.

To inactivate mature *C. jejuni* biofilms, we used bactericidal concentrations of phytochemicals and hypothesized that phytochemicals kill biofilm associated *C. jejuni* by potentially disrupting their cell membrane thereby leading to membrane dysfunction, cellular damage and inactivation of biofilms from the surfaces. *C. jejuni* biofilms were developed for 48 h since our TTC staining results suggest that *C. jejuni* biofilm matures by 48 h (Figure 1). We found that TC, EG and CR were effective in killing *C. jejuni* in the mature biofilms on polystyrene (Table 2) and steel surface (Table 3) at both temperatures. Previously, Lu et al. (2012) had reported inactivation of *C. jejuni* biofilms after 24 h treatment time with 1 μM concentration of diallyl sulfide (an antimicrobial agent from *Allum* spp). Antibiofilm efficacy of TC, EG and CR has also been reported against *L. monocytogenes* (Upadhyay et al., 2013) and *E. coli* (Perez-Conesa et al., 2006) suggesting that the phytochemicals exert antibiofilm effect on several pathogens, however, commonalities in their mechanism of action against various pathogens or the presence of a single target across pathogens that the plant compounds affect has not been identified yet. Considering these results, the select phytochemicals could be effective in reducing *C. jejuni* biofilm formation either in monoculture or when present with other biofilm forming foodborne pathogens; however, further experiments are needed to validate the results on multispecies biofilm of *C. jejuni*.

In the inactivation studies, 66.56 mM CR was the most effective in killing *C. jejuni* biofilms formed in the presence of chicken meat juice on polystyrene surfaces at both temperatures (Table 2). Similarly, in the presence of chicken meat juice, CR was the most effective followed by EG and TC in inactivating *C. jejuni* biofilm on stainless-steel coupons at 37°C in 1 min exposure time (Table 3). In general, we observed an increase in the antibiofilm effect of TC, EG and CR with an increase in their concentrations and more effective killing was found on biofilm developed at 20°C than at 37°C. The increased effectiveness of phytochemicals at 20°C could be due to weak attachment of metabolically active *C. jejuni* to surfaces at 20°C than at 37°C after 48 h as reflected by the absorbance value in TTC staining (Figure 1). Similar results were reported by Reeser et al. (2007) where the absorbance was five times lower at 25°C than at 37°C in the *C. jejuni* biofilms developed for 48 h. In our study, phytochemicals were more effective in reducing biofilms developed on steel surfaces (Table 3) than on polystyrene plates (Table 2) owing to good hydrophobicity of plastic surfaces for interaction with bacteria leading to stronger biofilm formation. Previously, Reeser et al. (2007) had determined that the physiochemical properties of the abiotic surfaces affect the *C. jejuni* attachment on surfaces to form biofilm and reported a higher degree of *C. jejuni* biofilm on hydrophobic surfaces (polystyrene and polyvinyl chloride) than on hydrophilic surfaces (glass, copper, and steel).

To validate the inactivation results, we visualized the architecture of treated biofilms using ESEM and CLSM. We observed that EPS was detached from bacterial cell surface leading to scattering of *C. jejuni* cells after 10 min of exposure to TC (18.91 mM), EG (15.22 mM), or CR (16.64 mM) (Figures 4A–D). Since EPS is critical for *C. jejuni* biofilms, loss of EPS could be a potential antibiofilm mechanism of the tested phytochemicals. In addition, predominant *C. jejuni* in the control were live (green) whereas the majority of *C. jejuni* were dead (red) after treatments (Figures 4a–d). Similar results of confocal microscopy were reported previously with TC, EG and CR against *L. monocytogenes* biofilms (Upadhyay et al., 2013).

In conclusion, TC, EG, and CR were effective in reducing *C. jejuni* biofilm formation and inactivating mature biofilms on polystyrene plates and stainless-steel coupons at 20 and 37°C. This reduction could potentially lead to reduced product contamination in processing plant. However, a correlation between a reduction in *C. jejuni* biofilm counts and corresponding reductions in pathogen load on carcass has not been conducted and could be a focus of future research. In addition, the effects of phytochemicals in monospecies biofilms of other *C. jejuni* strains as well as in multispecies *C. jejuni* biofilms should be evaluated in future studies. Proteomic analysis revealed select genes and proteins critical for biofilm formation were modulated by phytochemicals. However, further experiments are warranted to establish a correlation between changes in gene and corresponding protein expression in the biofilm.

## Data Availability

The raw data supporting the conclusions of this manuscript will be made available by the authors, without undue reservation, to any qualified researcher.

## Author Contributions

BW and AU designed the study. BW, IU, SS, KA, RL, and AU conducted the experiments. BW wrote the manuscript. AU, KV, DD, and AD critically analyzed and revised the manuscript.

## Disclaimer

Mention of a trade name, proprietary product, or specific equipment does not constitute a guarantee or warranty by the USDA and does not imply its approval to the exclusion of other products that may be suitable.

## Conflict of Interest Statement

The authors declare that the research was conducted in the absence of any commercial or financial relationships that could be construed as a potential conflict of interest.

## References

[B1] Adams T. B. Cohen S. M. Doull J. Feron V. J. Goodman J. I. Marnett L. J. (2004). The FEMA GRAS assessment of cinnamyl derivatives used as flavor ingredients. *Food Chem. Toxicol.* 42 157–185. 10.1016/j.fct.2003.08.021 14667463

[B2] Adams T. B. Cohen S. M. Doull J. Feron V. J. Goodman J. I. Marnett L. J. (2005). The FEMA GRAS assessment of hydroxy-and alkoxy-substituted benzyl derivatives used as flavor ingredients. *Food Chem. Toxicol.* 43 1241–1271. 10.1016/j.fct.2004.12.018 15950816

[B3] Annan-Prah A. Janc M. (1988). The mode of spread of campylobacter jejuni/coli to broiler flocks. *J. Vet. Med.* 35 11–18. 10.1111/j.1439-0450.1988.tb00461.x 3376623

[B4] Arita-Morioka K. I. Yamanaka K. Mizunoe Y. Ogura T. Sugimoto S. (2015). Novel strategy for biofilm inhibition by using small molecules targeting molecular chaperone DnaK. *Antimicrob. Agents Chemother.* 59 633–641. 10.1128/AAC.04465-14 25403660 PMC4291377

[B5] Asakura H. Yamasaki M. Yamamoto S. Igimi S. (2007). Deletion of peb4 gene impairs cell adhesion and biofilm formation in *Campylobacter jejuni*. *FEMS Microbiol. Lett.* 275 278–285. 10.1111/j.1574-6968.2007.00893.x 17714477

[B6] Bikandi J. Millán R. S. Rementeria A. Garaizar J. (2004). In silico analysis of complete bacterial genomes: PCR, AFLP–PCR and endonuclease restriction. *Bioinformatics* 20 798–799. 10.1093/bioinformatics/btg491 14752001

[B7] Birk T. Ingmer H. Andersen M. T. Jørgensen K. Brøndsted L. (2004). Chicken juice, a food-based model system suitable to study survival of *Campylobacter jejuni*. *Lett. Appl. Microbiol.* 38 66–71. 10.1046/j.1472-765x.2003.01446.x 14687218

[B8] Borges A. Abreu A. Dias C. Saavedra M. Borges F. Simões M. (2016). New perspectives on the use of phytochemicals as an emergent strategy to control bacterial infections including biofilms. *Molecules* 21:877. 10.3390/molecules21070877 27399652 PMC6274140

[B9] Boysen L. Nauta M. Rosenquist H. (2016). Campylobacter spp. and *Escherichia coli* contamination of broiler carcasses across the slaughter line in danish slaughterhouses. *Microb. Risk Anal.* 2 63–67. 10.1016/j.mran.2016.05.005

[B10] Bronnec V. Turňová H. Bouju A. Cruveiller S. Rodrigues R. Demnerova K. (2016). Adhesion, biofilm formation, and genomic features of *Campylobacter jejuni* Bf, an atypical strain able to grow under aerobic conditions. *Front. Microbiol.* 7:1002. 10.3389/fmicb.2016.01002 27446042 PMC4927563

[B11] Bronowski C. James C. E. Winstanley C. (2014). Role of environmental survival in transmission of *Campylobacter jejuni*. *FEMS Microbiol. Lett.* 356 8–19. 10.1111/1574-6968.12488 24888326

[B12] Brown H. L. Reuter M. Hanman K. Betts R. P. van Vliet A. H. (2015). Prevention of biofilm formation and removal of existing biofilms by extracellular DNases of *Campylobacter jejuni*. *PLoS One* 10:e0121680. 10.1371/journal.pone.0121680 25803828 PMC4372405

[B13] Brown H. L. Reuter M. Salt L. J. Cross K. L. Betts R. P. van Vliet A. H. (2014). Chicken juice enhances surface attachment and biofilm formation of *Campylobacter jejuni*. *Appl. Environ. Microbiol.* 80 7053–7060. 10.1128/AEM.02614-14 25192991 PMC4249011

[B14] Burt S. (2004). Essential oils: their antibacterial properties and potential applications in foods-a review. *Int. J. Food Microbiol.* 94 223–253. 10.1016/j.ijfoodmicro.2004.03.022 15246235

[B15] Byrd J. A. Hargis B. M. Caldwell D. J. Bailey R. H. Herron K. L. McReynolds J. L. (2001). Effect of lactic acid administration in the drinking water during preslaughter feed withdrawal on *Salmonella* and *Campylobacter* contamination of broilers. *Poult. Sci.* 80 278–283. 10.1093/ps/80.3.278 11261556

[B16] Čabarkapa I. Čolović R. Ðuragić O. Popović S. Kokić B. Milanov D. (2019). Anti-biofilm activities of essential oils rich in carvacrol and thymol against *Salmonella* enteritidis. *Biofouling* 35 361–375. 10.1080/08927014.2019.1610169 31088182

[B17] Castillo S. Heredia N. Arechiga-Carvajal E. García S. (2014). Citrus extracts as inhibitors of quorum sensing, biofilm formation and motility of *Campylobacter jejuni*. *Food Biotechnol.* 28 106–122. 10.1080/08905436.2014.895947

[B18] Castro A. G. Dorneles E. M. Santos E. L. Alves T. M. Silva G. R. Figueiredo T. C. (2018). Viability of *Campylobacter* spp. in frozen and chilled broiler carcasses according to real-time PCR with propidium monoazide pretreatment. *Poult. Sci.* 97 1706–1711. 10.3382/ps/pey020 29471351

[B19] Cody A. J. McCarthy N. D. van Rensburg M. J. Isinkaye T. Bentley S. Parkhill J. (2013). Real-time genomic epidemiological evaluation of human *Campylobacter* isolates by use of whole-genome multilocus sequence typing. *J. Clin. Microbiol.* 51 2526–2534. 10.1128/JCM.00066-13 23698529 PMC3719633

[B20] Dhillon A. S. Shivaprasad H. L. Schaberg D. Wier F. Weber S. Bandli D. (2006). Campylobacter jejuni infection in broiler chickens. *Avian. Dis.* 50 55–58. 10.1637/7411-071405r.1 16617982

[B21] Donlan R. M. (2002). Biofilms: microbial life on surfaces. *Emerg. Infect. Dis.* 8 881–890. 10.3201/eid0809.020063 12194761 PMC2732559

[B22] Donlan R. M. Costerton J. W. (2002). Biofilms: survival mechanisms of clinically relevant microorganisms. *Clin. Microbiol. Rev.* 15 167–193. 10.1128/cmr.15.2.167-193.2002 11932229 PMC118068

[B23] Dore M. H. (2015). “Threats to human health: use of chlorine, an obsolete treatment technology,” in *Global Drinking Water Management and Conservation*, ed. Dore M. H. (Cham: pringer), 197–212. 10.1007/978-3-319-11032-5_9

[B24] Fields J. A. Thompson S. A. (2008). *Campylobacter jejuni* CsrA mediates oxidative stress responses, biofilm formation, and host cell invasion. *J. Bacteriol.* 190 3411–3416. 10.1128/JB.01928-07 18310331 PMC2347403

[B25] García-Sánchez L. Melero B. Jaime I. Hänninen M. L. Rossi M. Rovira J. (2017). Campylobacter jejuni survival in a poultry processing plant environment. *Food Microbiol.* 65 185–192. 10.1016/j.fm.2017.02.009 28400001

[B26] Gradel K. O. Nielsen H. L. Schønheyder H. C. Ejlertsen T. Kristensen B. Nielsen H. (2009). Increased short-and long-term risk of inflammatory bowel disease after *salmonella* or *Campylobacter* gastroenteritis. *Gastroenterology* 137 495–501. 10.1053/j.gastro.2009.04.001 19361507

[B27] Hoffmann S. Batz M. B. Morris J. G. (2012). Annual cost of illness and quality-adjusted life year losses in the United States due to 14 foodborne pathogens. *J. Food Prot.* 75 1292–1302. 10.4315/0362-028X.JFP-11-417 22980013

[B28] Holley R. A. Patel D. (2005). Improvement in shelf-life and safety of perishable foods by plant essential oils and smoke antimicrobials. *Food Microbiol.* 22 273–292. 10.1016/j.fm.2004.08.006

[B29] Hwang S. Zhang Q. Ryu S. Jeon B. (2012). Transcriptional regulation of the CmeABC multidrug efflux pump and the KatA catalase by CosR in *Campylobacter jejuni*. *J. Bacteriol.* 194 6883–6891. 10.1128/JB.01636-12 23065977 PMC3510603

[B30] Jeong D. K. Frank J. F. (1994). Growth of *Listeria monocytogenes* at 10°C in biofilms with microorganisms isolated from meat and dairy processing environments. *J. Food Prot.* 57 576–586. 10.4315/0362-028x-57.7.576 31121716

[B31] Joshua G. P. Guthrie-Irons C. Karlyshev A. V. Wren B. W. (2006). Biofilm formation in *Campylobacter jejuni*. *Microbiology* 152 387–396. 10.1099/mic.0.28358-0 16436427

[B32] Kalmokoff M. Lanthier P. Tremblay T. L. Foss M. Lau P. C. Sanders G. (2006). Proteomic analysis of *Campylobacter jejuni* 11168 biofilms reveals a role for the motility complex in biofilm formation. *J. Bacteriol.* 188 4312–4320. 10.1128/jb.01975-05 16740937 PMC1482957

[B33] Kim J. S. Park C. Kim Y. J. (2015). Role of flgA for flagellar biosynthesis and biofilm formation of *Campylobacter jejuni* NCTC11168. *J. Microbiol. Biotechnol.* 25 1871–1879. 10.4014/jmb.1504.04080 26215271

[B34] Kim S. H. Park C. Lee E. J. Bang W. S. Kim Y. J. Kim J. S. (2017). Biofilm formation of *Campylobacter* strains isolated from raw chickens and its reduction with DNase I treatment. *Food Control* 71 94–100. 10.4014/jmb.1703.03052 28870004

[B35] Knowles J. R. Roller S. Murray D. B. Naidu A. S. (2005). Antimicrobial action of carvacrol at different stages of dual-species biofilm development by *Staphylococcus aureus* and *Salmonella enterica* serovar *typhimurium*. *Appl. Environ. Microbiol.* 71 797–803. 10.1128/aem.71.2.797-803.2005 15691933 PMC546778

[B36] Kovács J. K. Felsö P. Makszin L. Pápai Z. Horváth G. Ábrahám H. (2016). Antimicrobial and virulence-modulating effects of clove essential oil on the foodborne pathogen *Campylobacter jejuni*. *Appl. Environ. Microbiol.* 82 6158–6166. 10.1128/aem.01221-16 27520816 PMC5068167

[B37] Lehtola M. J. Pitkänen T. Miebach L. Miettinen I. T. (2006). Survival of *Campylobacter jejuni* in potable water biofilms: a comparative study with different detection methods. *Water. Sci. Technol.* 54 57–61. 10.2166/wst.2006.448 17037133

[B38] Letsididi K. S. Lou Z. Letsididi R. Mohammed K. Maguy B. L. (2018). Antimicrobial and antibiofilm effects of trans-cinnamic acid nanoemulsion and its potential application on lettuce. *LWT* 94 25–32. 10.1016/j.lwt.2018.04.018

[B39] Line J. E. (2001). Development of a selective differential agar for isolation and enumeration of *Campylobacter* spp. *J. Food Prot.* 64 1711–1715. 10.4315/0362-028x-64.11.1711 11726148

[B40] Lou Z. Letsididi K. S. Yu F. Pei Z. Wang H. Letsididi R. (2019). Inhibitive effect of eugenol and its nanoemulsion on quorum sensing–mediated virulence factors and biofilm formation by *Pseudomonas aeruginosa*. *J. Food Prot.* 82 379–389. 10.4315/0362-028X.JFP-18-196 30785306

[B41] Lu X. Samuelson D. R. Rasco B. A. Konkel M. E. (2012). Antimicrobial effect of diallyl sulphide on *Campylobacter jejuni* biofilms. *J. Antimicrobial. Chemother.* 8 1915–1926. 10.1093/jac/dks138 22550133 PMC3394439

[B42] Maisuria V. B. Lopez-de Los Santos Y. Tufenkji N. Déziel E. (2016). Cranberry-derived proanthocyanidins impair virulence and inhibit quorum sensing of *Pseudomonas aeruginosa*. *Sci. Rep.* 6:30169. 10.1038/srep30169 27503003 PMC4977528

[B43] Malik H. Rajagunalan S. Kumar M. S. Kataria J. L. Anjay P. Sachan S. (2017). Assessment of antibiotics effect on planktonic and biofilm forms of *Campylobacter Isolates*. *Israel J. Vet. Med.* 72:4.

[B44] Marder E. P. Cieslak P. R. Cronquist A. B. Dunn J. Lathrop S. Rabatsky-Ehr T. (2017). Incidence and trends of infections with pathogens transmitted commonly through food and the effect of increasing use of culture-independent diagnostic tests on surveillance-foodborne diseases active surveillance network, 10 US Sites, 2013-2016. *MMWR Morb. Mortal. Wkly. Rep.* 66 397–403. 10.15585/mmwr.mm6615a1 28426643 PMC5687182

[B45] McLennan M. K. Ringoir D. D. Frirdich E. Svensson S. L. Wells D. H. Jarrell H. (2008). *Campylobacter jejuni* biofilms up-regulated in the absence of the stringent response utilize a calcofluor white-reactive polysaccharide. *J. Bacteriol.* 190 1097–1107. 10.1128/jb.00516-07 17993532 PMC2223549

[B46] Melo R. T. Mendonça E. P. Monteiro G. P. Siqueira M. C. Pereira C. B. Peres P. A. (2017). Intrinsic and extrinsic aspects on *Campylobacter jejuni* biofilms. *Front. Microbiol.* 8:1332. 10.3389/fmicb.2017.01332 28769900 PMC5513903

[B47] Miyamoto K. N. Monteiro K. M. da Silva Caumo K. Lorenzatto K. R. Ferreira H. B. Brandelli A. (2015). Comparative proteomic analysis of *Listeria monocytogenes* ATCC 7644 exposed to a sublethal concentration of nisin. *J. Proteom.* 119 230–237. 10.1016/j.jprot.2015.02.006 25724729

[B48] Murphy C. Carroll C. Jordan K. N. (2006). Environmental survival mechanisms of the foodborne pathogen *Campylobacter jejuni*. *J. Appl. Microbiol.* 100 623–632. 10.1111/j.1365-2672.2006.02903.x 16553716

[B49] Naito M. Frirdich E. Fields J. A. Pryjma M. Li J. Cameron A. (2010). Effects of sequential *Campylobacter jejuni* 81-176 lipooligosaccharide core truncations on biofilm formation, stress survival, and pathogenesis. *J. Bacteriol.* 192 2182–2192. 10.1128/JB.01222-09 20139192 PMC2849453

[B50] Northcutt J. K. Smith D. P. Musgrove M. T. Ingram K. D. Hinton A. (2005). Microbiological impact of spray washing broiler carcasses using different chlorine concentrations and water temperatures. *Poult. Sci.* 84 1648–1652. 10.1093/ps/84.10.1648 16335135

[B51] Oh E. Jeon B. (2014). Role of alkyl hydroperoxide reductase (AhpC) in the biofilm formation of *Campylobacter jejuni*. *PLoS One* 9:e87312. 10.1371/journal.pone.0087312 24498070 PMC3909096

[B52] Oyarzabal O. A. (2005). Reduction of *Campylobacter* spp. by commercial antimicrobials applied during the processing of broiler chickens: a review from the United States perspective. *J. Food Prot.* 68 1752–1760. 10.4315/0362-028x-68.8.1752 21132992

[B53] Perez-Conesa D. McLandsborough L. Weiss J. (2006). Inhibition and inactivation of *Listeria monocytogenes* and *Escherichia coli* O157: H7 colony biofilms by micellar-encapsulated eugenol and carvacrol. *J. Food Prot.* 69 2947–2954. 10.4315/0362-028x-69.12.2947 17186663

[B54] Perkins D. N. Pappin D. J. Creasy D. M. Cottrell J. S. (1999). Probability-based protein identification by searching sequence databases using mass spectrometry data. *Electrophoresis* 20 3551–3567. 10.1002/(sici)1522-2683(19991201)20:18<3551::aid-elps3551>3.0.co;2-2 10612281

[B55] Pittman M. S. Elvers K. T. Lee L. Jones M. A. Poole R. K. Park S. F. (2007). Growth of *Campylobacter jejuni* on nitrate and nitrite: electron transport to NapA and NrfA via NrfH and distinct roles for NrfA and the globin Cgb in protection against nitrosative stress. *Mol. Microbiol.* 63 575–590. 10.1111/j.1365-2958.2006.05532.x 17241202

[B56] Qiu J. Feng H. Lu J. Xiang H. Wang D. Dong J. (2010). Eugenol reduces the expression of virulence-related exoproteins in *Staphylococcus aureus*. *Appl. Environ. Microbiol.* 76 5846–5851. 10.1128/AEM.00704-10 20639367 PMC2935054

[B57] Reeser R. J. Medler R. T. Billington S. J. Jost B. H. Joens L. A. (2007). Characterization of *Campylobacter jejuni* biofilms under defined growth conditions. *Appl. Environ. Microbiol.* 73 1908–1913. 10.1128/aem.00740-06 17259368 PMC1828834

[B58] Reuter M. Mallett A. Pearson B. M. van Vliet A. H. (2010). Biofilm formation by *Campylobacter jejuni* is increased under aerobic conditions. *Appl. Environ. Microbiol.* 76 2122–2128. 10.1128/AEM.01878-09 20139307 PMC2849235

[B59] Rockabrand D. Livers K. Austin T. Kaiser R. Jensen D. Burgess R. (1998). Roles of DnaK and RpoS in starvation-induced thermotolerance of *Escherichia coli*. *J. Bacteriol.* 180 846–854. 9473038 10.1128/jb.180.4.846-854.1998 PMC106963

[B60] Rosner B. M. Schielke A. Didelot X. Kops F. Breidenbach J. Willrich N. (2017). A combined case-control and molecular source attribution study of human *Campylobacter* infections in Germany, 2011–2014. *Sci. Rep.* 7:5139. 10.1038/s41598-017-05227-x PMC5505968 28698561

[B61] Siringan P. Connerton P. L. Payne R. J. Connerton I. F. (2011). Bacteriophage-mediated dispersal of *Campylobacter jejuni* biofilms. *Appl. Environ. Microbiol.* 77 3320–3326. 10.1128/AEM.02704-10 21441325 PMC3126433

[B62] Somers E. B. Schoeni J. L. Wong A. C. (1994). Effect of trisodium phosphate on biofilm and planktonic cells of *Campylobacter jejuni*, *Escherichia coli* O157: H7, *Listeria monocytogenes* and *Salmonella* typhimurium. *Int. J. Food Microbiol.* 22 269–276. 10.1016/0168-1605(94)90178-3 7986678

[B63] Spiller R. C. (2007). Role of infection in irritable bowel syndrome. *J. Gastroenterol.* 42 41–47. 10.1007/s00535-006-1925-8 17238025

[B64] Svensson S. L. Davis L. M. MacKichan J. K. Allan B. J. Pajaniappan M. Thompson S. A. (2009). The CprS sensor kinase of the zoonotic pathogen *Campylobacter jejuni* influences biofilm formation and is required for optimal chick colonization. *Mol. Microbiol.* 71 253–272. 10.1111/j.1365-2958.2008.06534.x 19017270 PMC2771394

[B65] Trachoo N. Frank J. F. (2002). Effectiveness of chemical sanitizers against *Campylobacter jejuni*–containing biofilms. *J. Food Prot.* 65 1117–1121. 10.4315/0362-028x-65.7.1117 12117244

[B66] Trachoo N. Frank J. F. Stern N. J. (2002). Survival of *Campylobacter jejuni* in biofilms isolated from chicken houses. *J. Food Prot.* 65 1110–1116. 10.4315/0362-028x-65.7.1110 12117243

[B67] Trevisan D. A. C. Silva A. F. D. Negri M. Abreu Filho B. A. D. Machinski Junior M. Patussi E. V. (2018). Antibacterial and antibiofilm activity of carvacrol against *Salmonella enterica* serotype *typhimurium*. *Braz. J. Pharm. Sci.* 54:e17229.

[B68] Turonova H. Briandet R. Rodrigues R. Hernould M. Hayek N. Stintzi A. (2015). Biofilm spatial organization by the emerging pathogen *Campylobacter jejuni*: comparison between NCTC 11168 and 81-176 strains under microaerobic and oxygen-enriched conditions. *Front. Microbiol.* 6:709. 10.3389/fmicb.2015.00709 26217332 PMC4499754

[B69] Upadhyay A. Arsi K. Wagle B. R. Shrestha S. Upadhyaya I. Bhargava K. (2017a). *In-Water Supplementation of Trans-Cinnamaldehyde Nanoemulsion Reduces Campylobacter jejuni Colonization in Broiler Chickens.* Available at: https://www.poultryscience.org/psa17/abstracts/40.pdf (accessed Feburary 28, 2019).

[B70] Upadhyay A. Arsi K. Wagle B. R. Upadhyaya I. Shrestha S. Donoghue A. M. (2017b). Trans-cinnamaldehyde, carvacrol, and eugenol reduce *Campylobacter jejuni* colonization factors and expression of virulence genes in Vitro. *Front. Microbiol.* 8:713. 10.3389/fmicb.2017.00713 28487683 PMC5403884

[B71] Upadhyay A. Upadhyaya I. Kollanoor-Johny A. Venkitanarayanan K. (2013). Antibiofilm effect of plant derived antimicrobials on *Listeria monocytogenes*. *Food Microbiol.* 36 79–89. 10.1016/j.fm.2013.04.010 23764223

[B72] Upadhyaya I. Upadhyay A. Arsi K. Liyanage R. Donoghue A. Rath N. (2017). *Plant-Derived Antimicrobial Eugenol Modulates C. jejuni Proteome and Virulence Critical for Colonization in Chickens.* Available at: https://www.poultryscience.org/psa17/abstracts/193.pdf (accessed Feburary 28, 2019).

[B73] Wagle B. R. Arsi K. Upadhyay A. Shrestha S. Venkitanarayanan K. Donoghue A. M. (2017a). β-resorcylic Acid, a phytophenolic compound, reduces *Campylobacter jejuni* in postharvest poultry. *J. Food Prot.* 80 1243–1251. 10.4315/0362-028X.JFP-16-475 28686495

[B74] Wagle B. R. Upadhyay A. Arsi K. Shrestha S. Venkitanarayanan K. Donoghue A. M. (2017b). Application of β-Resorcylic acid as potential antimicrobial feed additive to reduce *Campylobacter* colonization in broiler chickens. *Front. Microbiol.* 8:599. 10.3389/fmicb.2017.00599 28428779 PMC5382206

[B75] Wagle B. R. Upadhyay A. Arsi K. Upadhyaya I. Shrestha S. Venkitanarayanan K. (2017c). *Phytochemicals Reduce Biofilm Formation and Inactivates Mature Biofilm of Campylobacter jejuni.* Available at: https://www.poultryscience.org/psa17/abstracts/21.pdf (accessed Feburary 28, 2019).

[B76] Wagle B. R. Upadhyay A. Upadhyaya I. Arsi K. Shrestha S. Liyanage R. (2019). “Plant-derived antimicrobials modulate *Campylobacter jejuni* proteome essential for biofilm formation,” in *Proceedings of the Plant & Animal Genome conference XXVII*, San Diego, CA.

[B77] Whitchurch C. B. Tolker-Nielsen T. Ragas P. C. Mattick J. S. (2002). Extracellular DNA required for bacterial biofilm formation. *Science* 295 1487–1487. 10.1126/science.295.5559.1487 11859186

